# Response Letter to the Editor in Reference to Letter to the Editor about “Molecular Characterization of Animal *Fasciola* spp. Isolates from Kermanshah, Western Iran”

**Published:** 2018-08

**Authors:** Arezoo BOZORGOMID, Naser NAZARI, Eshrat BEIGOM KIA, Homa HAJJARAN, Mehdi MOHEBALI, Mojgan ARYAEIPOUR, Peyman HEIDARIAN, Mohammad Saeid EZATI, Mohamad Bagher ROKNI

**Affiliations:** 1. Dept. of Medical Parasitology and Mycology, School of Public Health, Tehran University of Medical Sciences, Tehran, Iran; 2. Dept. of Medical Parasitology and Mycology, School of Medicine, Kermanshah University of Medical Sciences, Kermanshah, Iran; 3. Center for Research of Endemic Parasites of Iran (CREPI), Tehran University of Medical Sciences, Tehran, Iran; 4. Dept. of Food Hygiene, Faculty of Veterinary Medicine, Science and Research Branch, Islamic Azad University, Tehran, Iran

## Dear Editor-in-Chief

We would like to thank Dr Lee for his interest in our paper and for taking time to express their concerns. We agree that chimeric sequences in eukaryotes like other prokaryotic organisms could result in false perceptions novel haplotype or species. Chimeras are artifact sequences formed by two or more biological sequences concatenated into a single one (1). The ability to detect chimeric sequences during PCR amplification of the 18S ribosomal RNA genes is critical to avoid from polluting the public databases (2).

Coding regions of rDNA in the phylogenetic studies of various organisms have been well studied in past decades (3, 4). The primary objective of our study was to identify of *Fasciola* species in Kermanshah Province, western Iran by PCR-RFLP of 18S, ITS1 and 5.8S rRNA genes. It should be noted, however, sequencing rRNA-ITS1 region was used to confirm the results of PCR-RFLP (5). While efforts to detect chimeric sequences in 16S rRNA have been made in the prokaryotic community, parallel efforts in the eukaryotic community have been underdeveloped (1). However, there are no comprehensive databases for better understanding of chimeras in eukaryotic organisms especially *Fasciola* parasites.
